# Sol-Gel Synthesis and Characterization of the Cu-Mg-O System for Chemical Looping Application

**DOI:** 10.3390/ma15062021

**Published:** 2022-03-09

**Authors:** Timofey M. Karnaukhov, Grigory B. Veselov, Svetlana V. Cherepanova, Aleksey A. Vedyagin

**Affiliations:** 1 Department of Materials Science and Functional Materials, Boreskov Institute of Catalysis SB RAS, 630090 Novosibirsk, Russia; karnaukhovtm@catalysis.ru (T.M.K.); g.veselov@catalysis.ru (G.B.V.); svch@catalysis.ru (S.V.C.); 2 Faculty of Natural Sciences, Novosibirsk State University, 630090 Novosibirsk, Russia; 3 Physical Faculty, Novosibirsk State University, 630090 Novosibirsk, Russia

**Keywords:** sol-gel synthesis, magnesium oxide matrix, copper oxide, chemical looping, reduction/oxidation cycling

## Abstract

A sol-gel technique was applied to prepare the two-component oxide system Cu-Mg-O, where MgO plays the role of oxide matrix, and CuO is an active chemical looping component. The prepared samples were characterized by scanning electron microscopy, low-temperature nitrogen adsorption, and X-ray diffraction analysis. The reduction behavior of the Cu-Mg-O system was examined in nine consecutive reduction/oxidation cycles. The presence of the MgO matrix was shown to affect the ability of CuO towards reduction and re-oxidation significantly. During the first reduction/oxidation cycle, the main characteristics of the oxide system (particle size, crystallization degree, etc.) undergo noticeable changes. Starting from the third cycle, the system exhibits a stable operation, providing the uptake of similar hydrogen amounts within the same temperature range. Based on the obtained results, the two-component Cu-Mg-O system can be considered as a prospective chemical looping agent.

## 1. Introduction

The catalysts based on copper and copper oxide are traditionally attractive for researchers and industrialists mainly due to their high activity in a number of industrially important processes at relatively low temperatures (about 100–300 °C). For instance, copper-containing systems were intensively studied in the water gas shift reaction [1,2,3,4,5,6,7,8] and steam reforming of methanol and methane [9,10,11,12]. In these cases, the main advantages of using copper as an active component are its low cost if compared with precious metals, high dispersity of copper species, and their strong interaction with the support that allows tuning the catalytic properties. Besides the mentioned applications, copper-based catalysts are highly efficient in the processes of total and partial (selective) oxidation [13,14,15,16,17,18,19,20,21,22,23,24,25], hydrogenation [26,27,28,29], dehydrogenation of alcohols [30,31,32,33], reduction of nitrogen oxides [34,35], etc.

Such a high activity of the copper-containing systems in the oxidation reactions is stipulated, in many ways, by the high reactive capacity of copper oxide CuO. As a rule, the reactions with its involvement are highly exothermic, while the kinetic parameters of the copper oxide reduction are quite advantageous. All this causes the increased attention to CuO as a prospective oxygen carrier for the developed chemical looping technologies, among which the chemical looping combustion processes [36,37,38] should be mentioned specially.

At present, it is not a secret that the size of the active component significantly affects its reactive capacity and, therefore, catalytic activity. In most cases, the higher the dispersion of the particles, the higher its activity. The Cu/CuO-containing systems are not an exception [39,40,41]. On the other hand, one of the main problems connected with these systems is their instability during the utilization at elevated temperatures. The copper nanoparticles have an addiction to their sintering and agglomeration [42]. This trend is more crucial in the case of nanocrystalline copper-based materials. Thus, the analysis of the melting behavior of copper gives the following predictions. If the bulk copper undergoes melting at the temperature of 1083 °C, then the copper particles of 20 nm in size should start to melt at ~1000 °C. Further decrease in particle size to 10 and 5 nm is expected to result in the melting temperature values of ~750 and ~500 °C. Therefore, an approaching of the process temperature to the expected melting temperature should lead to a noticeable increase in the lability of copper species and their agglomeration into the thermodynamically favorable large particles. In order to solve the mentioned problem, copper and its oxide are deposited on various supports, and the metal-support interactions play the key roles here, providing the long-term stability of the supported copper species during the catalyst exploitation [1,9,30,32,33,34,43,44,45].

To keep the high dispersity of the active component during the catalytic process, the small particles of this component should be uniformly distributed in the matrix of the support [46]. Aluminum and zirconium oxides are known to provide good enough thermal stability of the copper catalysts [34,47]. Moreover, alumina can form a number of joint phases with copper, including spinel materials exhibiting high activity in the redox reaction [48,49]. Similar effects are reported for the systems with iron oxide used as support [1]. In addition, the copper nanoparticles can be stabilized using zeolites. The latter facilitates the adsorption of the reagents, thus improving the catalytic activity [50]. Titanium oxide is less thermally stable; however, it provides the chemical stability of the copper catalysts towards poisoning [51,52,53]. Oppositely, carbonaceous supports possess high thermal stability and attractive mechanical and catalytic properties [30,32,54,55,56].

The stability of the copper-containing systems is principally defined by the preparation method. The sol-gel techniques give a number of advantages, including the mentioned small particle size and uniform distribution of one component within the matrix of another component serving as the support. Recently, such a sol-gel approach was successfully applied to prepare the two- and three-component oxide systems based on the MgO matrix [57,58,59,60]. Magnesium oxide is a unique material possessing attractive textural characteristics such as developed specific surface area and porosity. Its melting temperature is 2802 °C. Therefore, MgO is resistant to sintering even at significantly elevated process temperatures. All the mentioned properties provide the high thermal stability of the active components distributed within the MgO matrix. In addition, magnesium oxide can also form joint phases with the oxides of the majority of transition metals, thus, strengthening the metal-support interaction. This feature gives a broad spectrum of possibilities for controllable tuning of the catalytic and redox properties [61].

For the oxide systems obtained via the sol-gel approach, the preparation conditions significantly affect the characteristics of the final materials and, therefore, define their area of application. For instance, the amount of used alcohol influences the porosity and the agglomeration degree of the primary particles [62]. The next set of effects is connected to the applied alkaline agent, structure-directing surfactant, and calcination temperature [63,64,65,66]. These factors determine the textural and morphological features of the oxides. Among the advantages of sol-gel synthesis of two-component MgO-based systems, a uniform distribution of the second phase within the MgO matrix should be mentioned [65,66]. Such a distribution provides appropriate dispersity of the second phase. Thus, Barad et al. reported the suppression of the grain growth process by confining yttrium oxide nanocrystallites within a polycrystalline magnesium oxide [67]. On the other hand, the second component can interact with the matrix with the formation of new joint phases [65,68,69]. Both the size of the distributed particles and the presence of the joint phases govern the catalytic and optical properties [65,68,69,70,71].

In the present work, the oxide system Cu-Mg-O containing 15 wt% of CuO was synthesized by the sol-gel method. Recently, the effect of the CuO concentration on the textural properties was studied; the loading of 15 wt% was found to be an optimal value [72]. The copper salt-precursor was added at the stage of the gel formation. The performed investigation of the oxide system by low-temperature nitrogen adsorption, scanning electron microscopy, X-ray diffraction analysis, and temperature-programmed reduction/oxidation has revealed that the copper species are stabilized within the MgO matrix and keep their dispersity during the consecutive reduction/oxidation cycles.

## 2. Materials and Methods

### 2.1. Preparation of the Samples

#### 2.1.1. Sol-Gel Synthesis of Cu-Mg-OH and Cu-Mg-O Systems

The two-component xerogel (Cu-Mg-OH) and oxide (Cu-Mg-O) systems were obtained via a sol-gel technique. The piece of magnesium ribbon (1 g, purity of 99.9%, Sigma-Aldrich, St. Louis, MO, USA) was dissolved in 43 mL of methanol (Avantor Performance Materials, Gliwice, Poland). In order to stabilize the gel, toluene (Component-Reaktiv, Moscow, Russia) was added into the formed solution of magnesium methoxide. The methanol-to-toluene ratio was 1:1. Then, the solution was dropwise mixed with an aqueous solution of copper (II) nitrate (Baltic Enterprise, Saint-Petersburg, Russia). The obtained gel was dried at room temperature for 2 h. After an additional drying at 200 °C for 2 h, the xerogel sample was denoted as Cu-Mg-OH. Finally, the sample was calcined in the air at slow heating to 500 °C for 6 h. The obtained oxide was labeled as Cu-Mg-O. The resulting copper loading was 15 wt%, with respect to CuO.

#### 2.1.2. Preparation of Bulk CuO

The reference sample of bulk CuO was prepared by the thermal decomposition of copper (II) nitrate (Baltic Enterprise, Saint-Petersburg, Russia) in a furnace at slow heating to 500 °C for 6 h.

### 2.2. Characterization and Testing of the Prepared Samples

#### 2.2.1. Low-Temperature Nitrogen Adsorption

The specific surface area (SSA), pore volume (V_pore_), and average pore diameter (D_av_) of the samples were calculated from the low-temperature nitrogen adsorption data. The pore size distributions were obtained from the isotherms of nitrogen adsorption at 77 K using an ASAP-2400 (Micromeritics, Norcross, GA, USA) instrument. The measurement uncertainty of this method was ±3%.

#### 2.2.2. Differential Thermal Analysis (DTA)

The thermogravimetric (TG), differential thermogravimetric (DTG), and differential scanning calorimetry (DSC) profiles were registered using a Netzsch STA 409 PC/PG simultaneous thermal analyzer (NETZSCH-Gerätebau GmbH, Selb, Germany). The sample was heated in an inert atmosphere (nitrogen) within a temperature range of 25–700 °C with the ramping rate of 5 °C/min. The measurement uncertainty of this method was ±1%.

#### 2.2.3. Scanning Electron Microscopy (SEM)

The microscopic studies of the Cu-Mg-OH and Cu-Mg-O samples were performed using a JSM-6460 (JEOL Ltd., Tokyo, Japan) scanning electron microscope.

#### 2.2.4. Temperature-Programmed Reduction (H_2_-TPR)

The temperature-programmed reduction of the samples was carried out in a hydrogen flow. The gas mixture containing 10 vol% H_2_ in Ar was passed through the reactor with the sample with a flow rate of 40 mL/min. The temperature was increased from 20 to 900 °C with a ramping rate of 10 °C/min. Prior to the experiments, each sample was kept in an argon flow at 150 °C to remove the adsorbed water. The hydrogen concentration at the reactor outlet was measured using the standard thermal conductivity detector working at 70 mA. The instrument was calibrated by a direct method, by which the hydrogen-containing flow was controllably diluted with argon. The measurement uncertainty of this method was ±3%.

#### 2.2.5. Temperature-Programmed Reduction/Oxidation Cycling

The temperature-programmed reduction/oxidation cycling experiments were performed in a flow-through reactor system, which allows for regulating the gas flows and switching the reductive and oxidative gas mixtures in an automatic mode. The sample (200 mg) was loaded inside the quartz reactor. The reactor was purged with a nitrogen flow of 40 mL/min for 30 min and then fed with the reductive gas mixture containing 10 vol% H_2_ in N_2_ (total flow rate of 45.7 mL/min). The reactor was heated from 30 to 700 °C with a ramping rate of 10 °C/min. The hydrogen concentration in the outlet gas mixture was monitored using a hydrogen gas analyzer GAMMA-100 (FSUE “SPA “Analitpribor”, Smolensk, Russia). When the temperature reached 700 °C, the reactor was maintained at this temperature for 15 min and was cooled down to 30 °C in a nitrogen flow (40 mL/min). Then, the reactor inlet gas mixture was switched to an air flow (10 mL/min), and the reactor was heated to 500 °C with a ramping rate of 20 °C/min. After remaining at the final point for 30 min, the reactor was cooled down to 30 °C in an air flow, and the reactor inlet gas mixture was switched back to the reductive mixture. The described reduction/oxidation cycles were repeated nine times. The measurement uncertainty of this method was ±5%.

#### 2.2.6. In Situ X-ray Diffraction Analysis at the Temperature-Programmed Reduction/Oxidation Conditions

The X-ray diffraction (XRD) analysis of the Cu-Mg-O system was performed in an in-situ regime using a D8 diffractometer (Bruker, Karlsruhe, Germany). The reduction and oxidation procedures were carried out directly in the reactor chamber of the diffractometer. Initially, the sample was heated from 25 to 700 °C in hydrogen, cooled down in hydrogen, passivated in helium at 25 °C, and re-oxidized in a gas mixture containing 5 vol% O_2_ in helium at heating from 25 to 700 °C. Finally, the sample was cooled down to 25 °C in a helium flow. The temperature ramping rate was 10 °C/min. The gas flow rates were 20 mL/min. The registration of the XRD patterns was made within the 2θ range of 15–85° with a step of 0.05° and an accumulation time of 3 s at each temperature point (25, 300, 500, and 700 °C). The lattice parameters were defined from the patterns recorded at room temperature only (without thermal expansion). All the calculations were made using a TOPAS (Bruker, Karlsruhe, Germany) software based on the Rietveld method [73].

## 3. Results and Discussion

**Figure 1:**
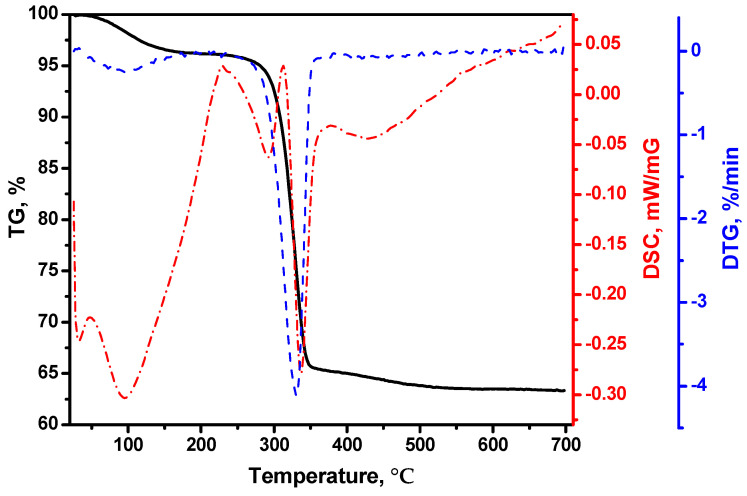
TG, DTG, and DSC profiles of the Cu-Mg-OH xerogel sample.

First, the as-prepared samples after drying at 200 °C (Cu-Mg-OH, xerogel) were studied by the DTA technique. Figure 1 presents the corresponding TG, DTG, and DSC profiles. It should be noted that the decomposition of bulk Cu(NO_3_)_2_ finishes at around 250 °C. In the case of dispersed particles, for example, when copper nitrate is supported on alumina, the complete decomposition can occur at even lower temperatures [74]. However, in the case of sol-gel-prepared systems, the residual nitrate species still can exist within the MgO matrix even after drying at 200 °C for 2 h. The first weight loss of about 3.8 wt% is observed below 200 °C and is attributed to the removal of the physically adsorbed water molecules. This process is accompanied by a large endothermal effect in the DSC curve. The main weight loss of 30.8 wt% is registered within a range of 240–360 °C. The presence of two endothermic effects in the DSC profile indicates that two processes take place consecutively. The decomposition of the residual species of copper nitrate is followed by the degradation of magnesium hydroxide. As a result, the second process occurs at lower temperatures if compared with the magnesium hydroxide systems reported in the literature [75,76]. As recently found [61], the intercalation of Cu^2+^ ions into the interlayer space of Mg(OH)_2_ simplifies the dehydration process. The last weight loss of 1.9 wt% is connected to the elimination of the residual species of organic molecules (toluene and methanol) [77].

**Figure 2:**
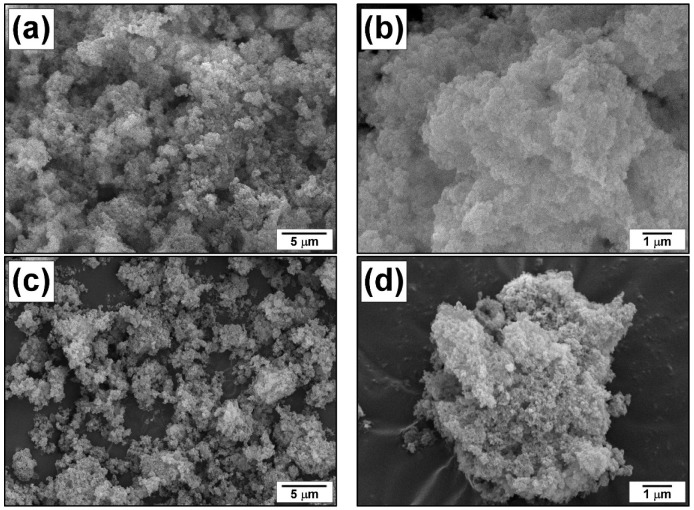
SEM images of the prepared samples: Cu-Mg-OH xerogel (**a**,**b**); Cu-Mg-O oxide (**c**,**d**).

The xerogel Cu-Mg-OH samples and the samples calcined at 500 °C (Cu-Mg-O, oxide) were examined by scanning electron microscopy. The obtained SEM images are shown in Figure 2. Both the samples are represented by poorly crystallized layered agglomerates consisting of nanosized primary particles. At the same time, the xerogel sample (Figure 2a,b) looks fluffy and lacy, while the oxide system (Figure 2c,d) seems to be more dense and compacted. It is natural to suppose that the initial xerogel with a developed structure undergoes collapsing under the calcination conditions.

**Figure 3:**
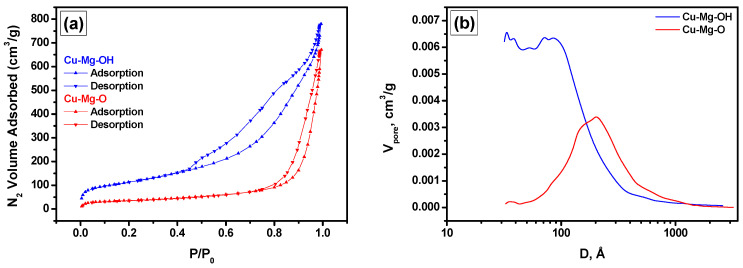
Low-temperature nitrogen adsorption/desorption isotherms (**a**) and pore size distribution (**b**) for the Cu-Mg-OH xerogel and Cu-Mg-O oxide sample.

The low-temperature nitrogen adsorption studies have revealed the changes in the porous structure that occurred during the calcination procedure. The nitrogen adsorption/desorption isotherms for the xerogel and oxide samples are presented in Figure 3a. Despite both the isotherms being characterized by the presence of a hysteresis loop, their shapes are significantly different. The quantitative parameters calculated from these results are summarized in Table 1. As seen, the SSA value drops down from 410 to 120 m^2^/g, i.e., more than three times, while the decrease in pore volume is not so crucial. An increase in the average pore size from 12 to 33 nm is connected with the shift of the maximum in pore size distribution towards larger sizes and the disappearance of the small pores (Figure 3b). It should be mentioned that the introduction of copper species inside the MgO matrix significantly worsens the textural properties of the latter. Thus, for pure MgO prepared via the same procedures with hydrolysis by distilled water instead of an aqueous solution of salt, the SSA value was as high as 243 m^2^/g [59].

**Figure 4:**
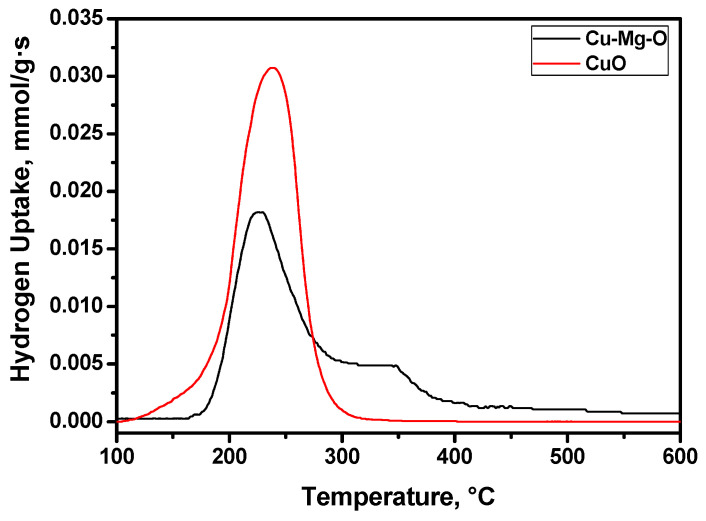
H_2_-TPR profiles for the bulk CuO oxide and prepared Cu-Mg-O system.

In order to investigate the effect of the MgO matrix on the reduction/oxidation behavior of copper, the prepared Cu-Mg-O sample was compared with the bulk CuO oxide by the H_2_-TPR method. Since the Cu-Mg-O system contains just 15 wt% of CuO, the hydrogen uptake intensities were normalized with respect to 1 g of CuO. Figure 4 demonstrates the resulting H_2_-TPR profiles. As evident, the profiles differ from each other. In the case of bulk CuO, its reduction takes place in a temperature range of 100–300 °C with a maximum at ~240 °C. The H_2_-TPR profile for the Cu-Mg-O sample has two hydrogen uptake peaks. The main peak appears at the lower temperature of 225 °C, thus, indicating the higher dispersity of the CuO particles if compared with the bulk copper oxide. The second uptake peak has a maximum at ~340 °C and gives a shoulder on the cumulative profile. This peak corresponds to the reduction in copper species strongly interacting with the matrix. In the case of the bulk Cu-Mg-O system, the whole reduction process occurs in a range of 160–400 °C.

**Figure 5:**
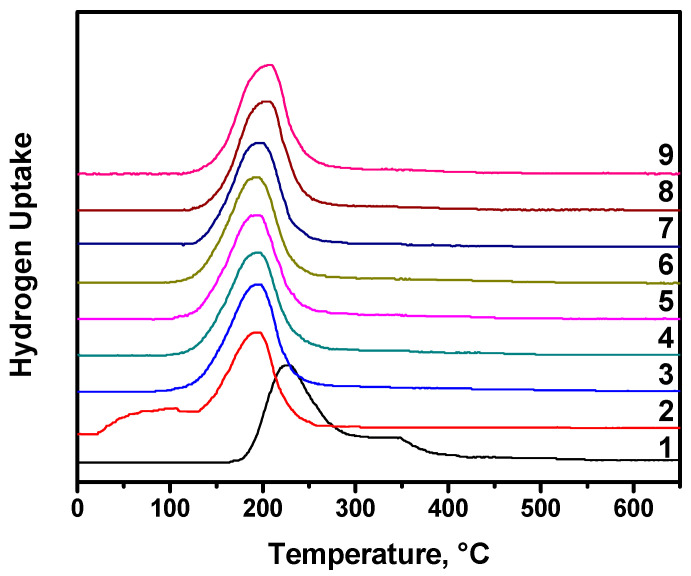
TPR profiles for the Cu-Mg-O system registered in nine consecutive reduction/oxidation cycles.

It should be remembered that, in the chemical looping concept, a cyclic regime of exploitation is considered. During these cycles, the properties of the oxygen carriers can be noticeably changed; the appropriate system should demonstrate a stable and reproducible behavior. In order to examine the efficiency and prospectivity of the prepared Cu-Mg-O system, it was tested in nine consecutive reduction/oxidation cycles. The obtained TPR profiles are shown in Figure 5. As was already discussed, the first reduction profile of the Cu-Mg-O system consists of two peaks, and the main uptake peak is shifted to lower temperatures if compared with the reference sample of bulk CuO (Figure 4). The second and subsequent reduction profiles of the two-component system are even more shifted to the left side (Figure 5). Moreover, the second reduction cycle is also represented by two uptake peaks, but the first of them has a maximum at ~75 °C. It can be supposed that these easily reducible copper particles are formed from the dispersed copper species that strongly interacted with MgO due to their reduction during the first reduction cycle. Already in the third reduction cycle, this low-temperature peak is disappeared. Note that the total hydrogen uptake is almost constant in all the cycles. The profiles from the third to ninth cycles show no remarkable difference. At the same time, the position of the main uptake peak slightly shifts towards high temperatures from 194 °C for the first cycle to 204 °C for the ninth cycle. It can be concluded here that the MgO matrix provides the thermal stabilization of the dispersed copper species. Just a small sintering effect is observed under the redox cycling conditions.

**Figure 6:**
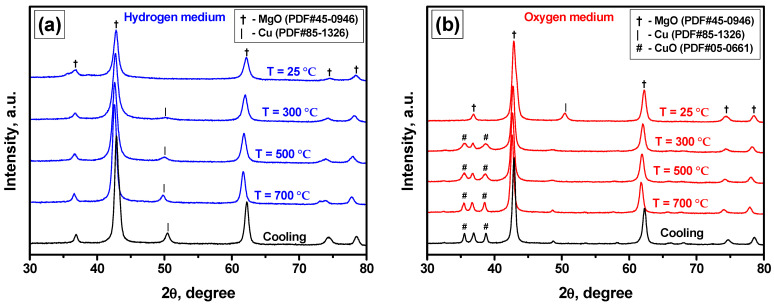
XRD patterns of the Cu-Mg-O system recorded in an in-situ regime at hydrogen (**a**) and oxygen (**b**) atmospheres.

At the final stage of the research, the Cu-Mg-O system was studied by an in-situ XRD technique. Figure 6 illustrates the corresponding XRD patterns recorded at 25, 300, 500, and 700 °C under reductive (Figure 6a) and oxidative (Figure 6b) conditions. The quantitative parameters obtained from the XRD data are collected in Table 2.

According to the presented patterns, for the initial sample, only the reflections at ~36, 43, 62, 74, and 78 degrees corresponding to the magnesium oxide phase are detected (Figure 6a). Supposedly, all the copper species are in a roentgen-amorphous state. These species undergo reduction with the temperature rise. The corresponding reflection at ~50 degrees appears already at 300 °C, and its intensity grows with the further temperature increase, indicating the formation of metallic copper nanocrystallites. The average size of the formed metallic copper particles is estimated to be 10 nm (Table 2). The MgO phase also undergoes crystallization, and the reflections become narrow and more intensive. Therefore, the initially observed poorly crystalline structure (see Figure 2) transforms into a nanocrystalline one. The ordering of the oxide structure is accompanied by the enlargement of the primary particles.

The oxidative part of the redox cycle oppositely shows the disappearance of the Cu(0) phase and the formation of the CuO phase (Figure 6b). The corresponding reflections of the oxide phase are seen at ~35 and 38 degrees starting from 300 °C. After 700 °C, they seem to be well-crystallized, with an average size of ~25 nm. These results confirm the previously made assumption concerning the subsequent formation of Cu(0) and CuO phases from the roentgen-amorphous well-dispersed copper species that strongly interacted with the MgO matrix. As follows from the data presented in Table 2, the lattice parameter of the initial sample is enlarged if compared with the conventional magnesium oxide (a = 4.211 Å). After the reduction/oxidation cycle, this parameter approximates the standard value that testifies to the exit of the copper species from the MgO matrix.

## 4. Conclusions

In modern industry, the oxides of transition metals are widely applied in chemical looping processes. However, being used in the bulk form, they undergo rapid agglomeration and sintering that significantly diminishes their efficiency in the redox cycles. Therefore, the use of an inert oxide matrix that preserves the high dispersity of the active component is an actual task. In the present work, the two-component Cu-Mg-O system was synthesized by the sol-gel method. The obtained material possesses a layered mesoporous structure with the developed surface area. The redox behavior of the two-component oxide system differs from that for the bulk CuO reference sample. In the initial state, the copper species are roentgen-amorphous and partly exhibit a strong interaction with the MgO matrix. During the first reduction/oxidation cycle, the final formation of the phase composition of the system takes place. The formed CuO nanoparticles undergo reproducible reduction to metallic Cu(0) nanoparticles with a maximum of hydrogen uptake at near 200 °C. Due to the presence of the MgO matrix, the dispersity of the CuO/Cu(0)-active species remains the same during the redox cycling. Therefore, this system can be considered as a prospective chemical looping material.

## Figures and Tables

**Table 1:** Low-temperature nitrogen adsorption data for the Cu-Mg-OH xerogel and Cu-Mg-O oxide samples.

Sample	SSA, m^2^/g	V_pores_, cm^3^/g	D_av_, nm
Cu-Mg-OH	410 ± 12	1.23 ± 1.1	12 ± 2
Cu-Mg-O	120 ± 4	1.04 ± 1.0	33 ± 4

**Table 2:** Quantitative characteristics (average size of crystallites <D>; the lengths a, b, and c of the three cell edges meeting at a vertex, and the angle β between edges a and c) obtained from the XRD data.

Phase (wt%)	Initial		After Reduction		After Oxidation	
	a, Å	<D>, nm	a, Å	<D>, nm	Lattice Parameter	<D>, nm
MgO	4.222(1)	8	4.220(1)	12	a = 4.216(1) Å	16
Cu (13%)	-	-	3.621(1)	10	-	-
CuO (6%)	-	-	-	-	a = 4.691(2) Å b = 3.423(1) Å c = 5.137(2) Å β = 99.42(3) °	25

## Data Availability

The data presented in this study are available on request from the corresponding author.
